# Pearl millet response to drought: A review

**DOI:** 10.3389/fpls.2023.1059574

**Published:** 2023-02-10

**Authors:** Nikee Shrestha, Hao Hu, Kumar Shrestha, Andrew N. Doust

**Affiliations:** ^1^ Department of Plant Biology, Ecology and Evolution, Oklahoma State University, Stillwater, OK, United States; ^2^ Center for Plant Science Innovation and Department of Agronomy and Horticulture, University of Nebraska-Lincoln, Lincoln, NE, United States

**Keywords:** drought, pearl millet, RNA-Seq, tillers, QTL, abiotic stress

## Abstract

The C4 grass pearl millet is one of the most drought tolerant cereals and is primarily grown in marginal areas where annual rainfall is low and intermittent. It was domesticated in sub-Saharan Africa, and several studies have found that it uses a combination of morphological and physiological traits to successfully resist drought. This review explores the short term and long-term responses of pearl millet that enables it to either tolerate, avoid, escape, or recover from drought stress. The response to short term drought reveals fine tuning of osmotic adjustment, stomatal conductance, and ROS scavenging ability, along with ABA and ethylene transduction. Equally important are longer term developmental plasticity in tillering, root development, leaf adaptations and flowering time that can both help avoid the worst water stress and recover some of the yield losses *via* asynchronous tiller production. We examine genes related to drought resistance that were identified through individual transcriptomic studies and through our combined analysis of previous studies. From the combined analysis, we found 94 genes that were differentially expressed in both vegetative and reproductive stages under drought stress. Among them is a tight cluster of genes that are directly related to biotic and abiotic stress, as well as carbon metabolism, and hormonal pathways. We suggest that knowledge of gene expression patterns in tiller buds, inflorescences and rooting tips will be important for understanding the growth responses of pearl millet and the trade-offs at play in the response of this crop to drought. Much remains to be learnt about how pearl millet’s unique combination of genetic and physiological mechanisms allow it to achieve such high drought tolerance, and the answers to be found may well be useful for crops other than just pearl millet.

## 1 Introduction

C4 millets, including foxtail millet (S*etaria italica*), barnyard millet (*Echinochloa frumantacea* and *E. esculenta*), proso or common millet (*Panicum milaceum*), and pearl millet (*Cenchrus americanus*) are extremely drought tolerant, and are relied on by farmers in marginal growing environments in many regions of the world (Doust et al., 2009; Li and Brutnell, 2011; Renganathan et al., 2020; Pardo and VanBuren, 2021; Satyavathi et al., 2021). Pearl millet is one of the most tolerant, being domesticated from its wild progenitor, *Cenchrus americanus* ssp.*violaceum* in the Sahel region of West Africa (Oumar et al., 2008; Burgarella et al., 2018), where annual rainfall varies between 200 to 600 mm (Bidinger et al., 1987). In this region, growing areas are characterized by a long dry season and a highly variable and short rainy season, conditions where few other grain crops would consistently yield, or even survive (Debieu et al., 2018). Several pearl millet accessions have been found from even drier regions, including areas in the Mauritania and Mali regions with average rainfall less than 50 mm (Harlan, 1975; Pucher et al., 2015). Because of its drought-hardiness, pearl millet is pre-adapted for the predicted increased desertification that will result from climate change (Council, 2010), and has behavioral responses to drought damage that preserve at least some yield for the farmer. Recent breeding efforts have been directed at increasing yield and decreasing susceptibility to diseases (Serba et al., 2017), with a concomitant change in the architecture of plants from many-tillered to few, and for inflorescences on those few tillers to be much larger and higher-yielding. This so-called ideal architecture (Jiao et al., 2010) is most efficient under more domesticated growing environments, with adequate water and soil nutrients, but may be less efficient under typical subsistence agriculture with uncertain water supplies. Pearl millet has the potential to play an important role in feeding the expanding world population, predicted to reach 9.1 billion by 2050, where, to meet the food demand of this huge population, cereal production needs to increase from 2.1 billion to up to 3 billion tons (Alexandratos, 2009). Making better use of marginal lands for crop cultivation will be an important component in increasing food production, and pearl millet will be a useful crop in these areas, as well as offering many lessons in tolerating drought that may be able to be translated to other crops. Importantly, breeders need to take the drought responses of pearl millet in the landscape in which it is best adapted into consideration in their breeding efforts.

Studies of the effects of drought on pearl millet include genetic analyses of variation in flowering time (Yadav et al., 2004), tillering (Mahalakshmi et al., 1987), grain yield (Bidinger et al., 1987; Yadav et al., 2002; Yadav et al., 2004), biochemical analyses (Choudhary et al., 2021), osmolyte analysis (Kusaka et al., 2005b), proteomic analysis (Ghatak et al., 2016) and gene expression analysis (Choudhary and Padaria, 2015; Dudhate et al., 2018; Jaiswal et al., 2018; Shivhare et al., 2020a; Shivhare et al., 2020b). The application of drought stress varies radically between these studies, from simulating drought using osmotically stressing chemicals to field trials. This review analyzes the characteristics of pearl millet which foster drought resistance, including both short- and longer-term responses to drought. The great variation in pearl millet accessions that is available, as well as the feasibility of introgressing desirable characteristics from wild species, results in a huge range of possibilities for engineering drought tolerance/resistance in pearl millet. We provide an overview of the morphological, physiological, and biochemical mechanisms pearl millet uses to avoid or tolerate drought stress, a detailed analysis of what we have learnt from quantitative trait locus (QTL) analysis and transcriptomic studies, and provide recommendations about where effort might best next be placed.

## 2 Pearl millet responses to drought resistance

**Figure 1:**
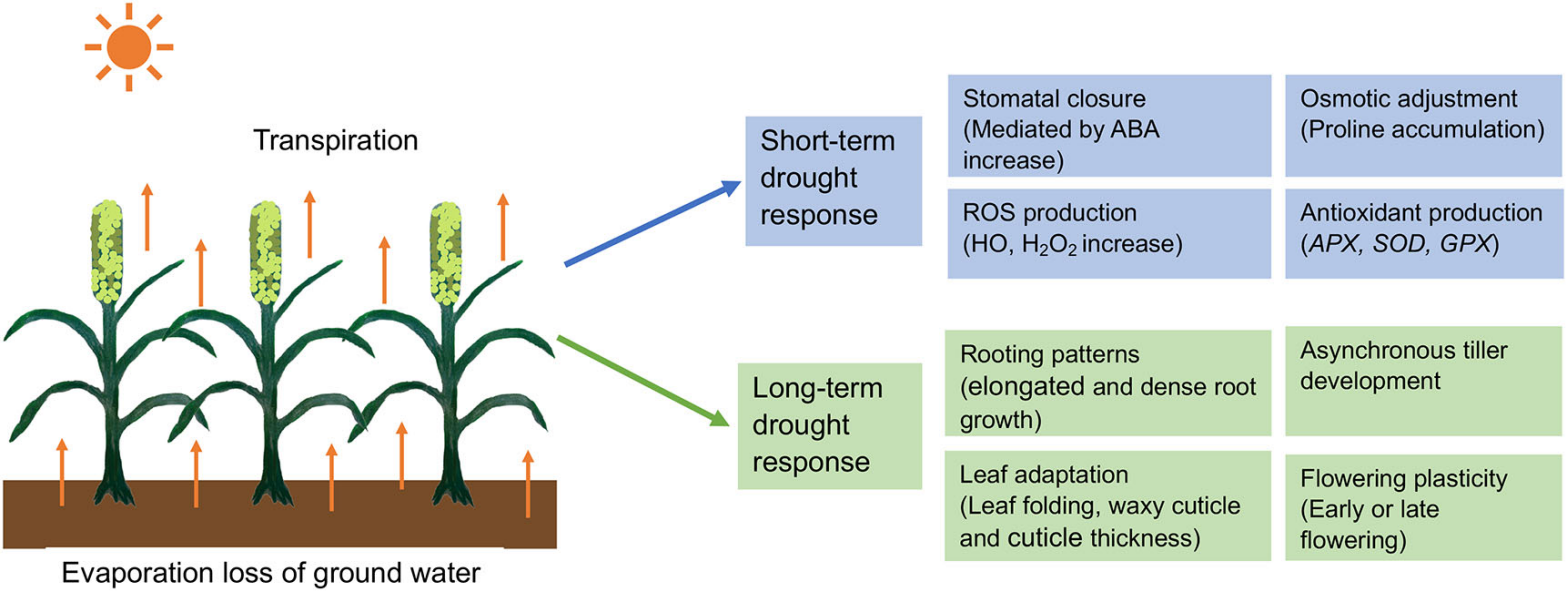
Short-term and long-term responses of pearl millet to adapt drought stress. ROS, Reactive oxygen species; HO, Hydroxyl radical; H_2_O_2_, Hydrogen peroxide; APX, *Ascorbate peroxide; GPX, Glutathione peroxidase; SOD, Superoxide dismutase*.

In pearl millet, drought studies have been conducted in each of the three main phases: vegetative, panicle development, and grain-filling (Shivhare et al., 2020a) and these studies have identified resistance mechanisms involving drought avoidance, tolerance, escape, and recovery. These mechanisms can be grouped into short- and long-term responses, as we discuss below (**Figure 1**). In addition, pearl millet is a C4 grass using the NADP-dependent malic enzyme (NADP-ME) pathway and is thus already preadapted for efficient water use in high light environments (Pardo and VanBuren, 2021). C4 grasses initially fix CO2 in the mesophyll cells to make a 4-carbon sugar that is then transported to specialized bundle sheath cells, where the CO2 is released before entering the Rubisco C3 pathway to make glucose. The light reactions of photosynthesis also occur preferentially in the mesophyll cells, rather than the bundle sheath cells, thus decoupling oxygen evolution *via* the splitting of water from carbon fixation *via* the Rubisco pathway, reducing competition between oxygen and CO_2_ molecules for the Rubisco enzyme. In practical terms, this increases water use efficiency by allowing C4 plants to have less need for gas exchange to get rid of excess oxygen, reducing the amount of time that stomates need to be open for gas exchange and thus for water loss, compared to C3 grasses like wheat or rice.

### 2.1 Short term responses

#### 2.1.1 Stomatal conductance

Stomata are vital to the plant’s existence, as they provide passage for gas and water exchange to conduct photosynthesis and transpiration (Li et al., 2017). Generally, stomatal closure is highly influenced by environmental conditions and is the first step for avoiding water loss in water stressed conditions, but plants do it at the expense of gaining carbon dioxide for carbon fixation. Stomatal conductance in pearl millet has been implicated in drought tolerance, as a major QTL for drought tolerance on linkage group 2 co-localizes with one of the QTL for lower transpiration rate under drought stress (**Tables 1**, **S1**) (Aparna et al., 2015; Tharanya et al., 2018).

**Table 1:** Summary of QTLs identified associated with yield component traits during drought stress caused at various stages of growth period across the seven linkage groups from various studies.

Drought stress imposed at vegetative stage	Drought stress imposed at reproductive stage
**Biomass production:** LG 1 and 4 (Kholová et al., 2012; Aparna et al., 2015) LG 2 (Kholová et al., 2012; Aparna et al., 2015; Debieu et al., 2018) LG 3 (Debieu et al., 2018) LG 6 (Kholová et al., 2012; Debieu et al., 2018) LG 7 (Kholová et al., 2012; Aparna et al., 2015) **Leaf dry weight** LG 2, 6 and 7 (Aparna et al., 2015) **Leaf area** LG 2, 5 and 6 (Aparna et al., 2015) **Specific leaf weight** LG 1, 2 and 5 (Aparna et al., 2015) **Stem dry weight** LG 1, 2 and 5 (Aparna et al., 2015) **Tiller number** LG 1 and 4 (Aparna et al., 2015) **Shoot weight** LG 2 and 5 (Aparna et al., 2015) **Root dry weight** LG 2 and 7 (Aparna et al., 2015) **Transpiration rate under low vapor pressure deficit (VPD)** LG 2 and 7 (Kholová et al., 2012) LG 3 (Kholová et al., 2012; Aparna et al., 2015) **Transpiration rate under high VPD** LG 1, 4 and 6 (Aparna et al., 2015) LG 2 (Kholová et al., 2012; Aparna et al., 2015) LG 7 (Kholová et al., 2012) **Transpiration under low VPD** LG 2 and 3 (Kholová et al., 2012) LG 6 (Aparna et al., 2015) LG 7 (Kholová et al., 2012; Aparna et al., 2015) **Transpiration under high VPD** LG 2 and 7 **(** Kholová et al., 2012; Aparna et al., 2015 **)** LG 1, 5 and 6 (Aparna et al., 2015) LG 3 (Kholová et al., 2012)	**Biomass production** LG 2 (Yadav et al., 2002) **Grain yield** LG 1 and 2 (Yadav et al., 2002; Yadav et al., 2004; Bidinger et al., 2007) LG 3 and 4 (Bidinger et al., 2007) LG 5 (Yadav et al., 2004) LG 7 ((Yadav et al., 2004; Bidinger et al., 2007) **Grain mass** LG 2 (Yadav et al., 2002; Bidinger et al., 2007) **Harvest index** LG 2 (Yadav et al., 2002; Yadav et al., 2004; Bidinger et al., 2007) LG 6 (Yadav et al., 2002; Yadav et al., 2004) LG 3, 5 and 7 (Yadav et al., 2004; Bidinger et al., 2007) **Panicle harvest index** LG 1 (Yadav et al., 2004; Bidinger et al., 2007) LG 5 and 7 (Yadav et al., 2004) LG 2, 3 and 6 (Bidinger et al., 2007) **Flowering time** LG 2, 3, 4 and 6 (Yadav et al., 2002; Yadav et al., 2004) **Stover yield** LG 2 and 6 (Yadav et al., 2002; Yadav et al., 2004) LG 4 (Yadav et al., 2002) LG 5 and 7 (Yadav et al., 2004) **Panicle grain number** LG 1 and 6 (Yadav et al., 2002) **Panicle number** LG 6 (Yadav et al., 2002)

Text above each single box informs about the stage where drought was imposed in the population.

Stomatal closure is mediated by accumulation of ABA; this acts as the first signal for drought response to prevents water loss and improve water use efficiency (Henson et al., 1981b). Kholova et al. (2010) reported that the ABA levels of the drought tolerant accession 863B-P3 had significantly increased levels of ABA at the vegetative stage. Transcriptomic analysis of pearl millet genotype *Tifleaf 3* during the vegetative stage, after 48 hours of drought treatment, revealed significant expression of abscisic acid related genes such *pyrabactin resistance (PYR)-like (PYL)* gene family (Sun et al., 2020). *PYL* are receptor for ABA which interacts with *protein phosphatases* (*PP2C)* and *SNF1-related* protein kinase (*SnRK2s)* to modulate stomatal closure (Zhang et al., 2021). Pearl millet genotypes having high leaf water potential were found to accumulate a significant amount of ABA in water deficit conditions compared to ones having lower leaf water potential (Henson et al., 1981a).

#### 2.1.2 Osmotic adjustment

Osmotic adjustment is a major drought tolerant mechanism in plants to maintain cell turgor, relative water content, cell expansion, photosynthesis and continued stomatal conductance (Jones and Turner, 1980; Henson et al., 1982). Plants accumulate organic and inorganic cellular components as osmolytes for lowering osmotic potential. In field conditions, pearl millet has been reported to adjust its osmotic potential successfully in response to water stress (Henson et al., 1982), in a manner similar to other drought tolerant C3 and C4 grasses, such as upland rice (Lum et al., 2014), wheat genotypes (Hong-Bo et al., 2006), and sorghum (Blum and Ebercon, 1976). The biochemical basis of osmotic adjustment has both inorganic as well as organic components, with the association of proline accumulation under water stress being associated with increased drought tolerance (Vijayalakshmi et al., 2012; Shivhare and Lata, 2017). The relative importance of different osmotic components may vary between genotype and life stage, with plants at the vegetative stage accumulating comparatively larger amounts of inorganic components such as K+ and NO3- compared to organic components such as proline, amino acids, and soluble sugars (Kusaka et al., 2005b).

Osmotic adjustment is particularly difficult when plants are stressed by high salinity. Six QTL-NILs associated with drought from accessions H 77/833-2, PRLT 2/89-33, and the mapping population formed from a cross between these, were subjected to a wide range of salinity and alkalinity stress at 24 days after sowing (Sharma et al., 2011). They reported that the drought susceptible parent H77/833-2 accumulated a significant concentration of toxic Na+ under stress treatments compared to drought tolerant NILs and the drought tolerant parent, PRLT 2/89-33. Their results suggest that the drought tolerant QTLs limit Na+ transportation, lower transpiration rates and thereby improve drought tolerance (Sharma et al., 2011). These QTL-NILs were then studied under drought stress at the flowering stage, and found that drought tolerant NILs performed better by differentially regulated Na+ accumulation in roots, and limiting translocation of Na+ to younger leaves, along with osmotic adjustment of leaf water potentials by high proline accumulation to restrict Na+ influx (Sharma et al., 2014).

#### 2.1.3 Antioxidant defense and ROS scavenging ability

Reactive oxygen species (ROS) such as singlet oxygen (^1^O_2_), superoxide radical (O_2-_), hydroxyl radical (HO) and hydrogen peroxide (H_2_O_2_) are known to be produced during biotic and abiotic stresses (Dudhate et al., 2018). However, plants rapidly produce different forms of ROS and scavenge them simultaneously to balance between defense signaling mechanisms and ROS toxicity (Qi et al., 2017). The accumulation of ROS can cause oxidative stress toxicity which causes lipid peroxidation and protein denaturation, causing significant tissue damage (Anjum et al., 2017). To avoid oxidative damage in plants during stress, resistant plants produce antioxidants to detoxify oxidative stress either by scavenging ROS superoxide or by activating various detoxifying and defensive proteins (Mates, 2001).

Several studies on expression profiling of antioxidant genes have been conducted in pearl millet under drought stress, and drought tolerant genotypes of pearl millet treated with polyethylene glycol simulating drought stress at early and late seedling stage showed significant increases in genes encoding *ascorbate peroxide (APX)*, *glutamyl-tRNA reductase* (*GlutR)* and *superoxide dismutase (SOD)* (Shivhare and Lata, 2017). The increased expression of genes encoding these three enzymes in a tolerant pearl millet genotype suggests their role in ROS scavenging during drought stress (Shivhare and Lata, 2019). Such conclusions were reached also in a transcriptomics analysis of pearl millet treated during the vegetative stage with drought that revealed the significant up-regulation of DEGs encoding ROS scavenging enzymes such as *SOD, APX* and *glutathione peroxidase (GPX)* (Sun et al., 2020). However, in another study that contrasted lines with and without a major terminal drought QTL, there was no major changes in enzyme activity except for an increase in APX5 (Kholová et al., 2011), leading the authors of that study to speculate that an increase in antioxidant enzymes might not play a direct role in terminal drought tolerance of pearl millet but rather help to minimize ROS toxicity in plants (Kholová et al., 2011).

### 2.2 Long term responses

#### 2.2.1 Rooting patterns

The development of elongated deep roots enables plants to take up more water from deeper soil horizons to maintain high leaf water potential in drought conditions, while not altering the physiological and biochemical status of the plant (Ekanayake et al., 1985; Zegada-Lizarazu and Iijima, 2005). The embryonic root system in pearl millet consists of the primary root, whose maximum vertical growth occurs during the first week after germination. During this early period, the primary root exhibits rapid elongation (9.1 cm per day), faster than other crops such as maize and wheat (2.7 cm per day) (Passot et al., 2016), allowing it to rapidly seek out moister subsurface soil. After approximately a week, lateral roots start to initiate on the primary root, and adventitious crown roots emerge from the base of the shoot. Primary roots are characterized by a large metaxylem vessel, whereas crown roots are thicker with a significantly larger stele that contains more than one metaxylem. The rate of elongation of the primary root and the density of lateral roots along the primary root varies with genotype (Passot et al., 2016), suggesting potential for breeding for elongated root systems during seedling establishment. Lateral root types differ between genotypes and may offer a further opportunity for breeding for dense root systems capable of efficiently using available ground water (Passot et al., 2016).

Under drought conditions, pearl millet exhibits both elongated and dense root growth systems, and studies have shown that the capacity for water uptake by pearl millet from wet subsoil layers through an increase in lateral root density increased when the drought treatment was imposed on the topsoil (Zegada-Lizarazu and Iijima, 2005). In comparison with other species of millet, drought imposed on the topsoil layer increased water uptake and resulted in larger leaf area and higher shoot biomass in drought resistant genotypes of pearl millet (Zegada-Lizarazu and Iijima, 2005). The deep roots of pearl millet were also found to be able to penetrate compacted soil layers in drought-stressed conditions. Within pearl millet, those genotypes which had lower osmotic adjustment to drought stress were reported to have longer total root lengths (Kusaka et al., 2005a), presumably as a result of the search to find water. Root architecture and plasticity play an important role in crop growth in drought-prone environments, yet there has been relatively little study on root development due to difficulty in phenotyping this trait. However, the development of models for estimating root length density in pearl millet and other crops such as maize will be important in advancing our knowledge of this vital trait (Das et al., 2015; Faye et al., 2019; Shao et al., 2021).

#### 2.2.2 Leaf adaptations

Under drought stress, pearl millet will allow its leaves to fold and roll, or even wither, in order to decrease leaf canopy area and reduce transpiration (Redmann, 1985; Kadioglu and Terzi, 2007). Leaf withering is especially apparent during drought stress at the grain-filling stage (Do et al., 1996). A pearl millet accession known for its drought resistance (IP8210) exhibited leaf folding due to a drought imposed at 21 days after gemination, yet recovered after re-watering at 12 days after drought initiation (Kusaka et al., 2005a). QTL studies have identified a QTL on linkage group 2 that is associated with leaf rolling during drought study, and this QTL colocalizes with QTL that have been identified for drought resistance from previous studies (Yadav et al., 2002; Yadav et al., 2004; Bidinger et al., 2007; Kholová et al., 2012; Aparna et al., 2015; Debieu et al., 2018) (**Tables 1**, **S1**).

Studies exploring stomatal density differences among pearl millet lines have reported variability in stomatal frequencies among genotypes (Teowolde et al., 1986). However, when differences in stomatal density were investigated among near isogenic lines, either with or without introgressed terminal drought QTL, along with the tolerant and susceptible parental lines, no differences were found (Kholova et al., 2010). Such results may be specific to the genotypes examined, but it suggests that more investigation into the role of stomatal density in drought response is warranted.

Another drought resistance mechanism is provided by the presence of a waxy cuticle (Bi et al., 2017), and, among the predicted 38,579 genes in the pearl millet genome assembly, there is a huge enrichment of genes found involved in cutin, flavonoid, suberin and wax biosynthesis (Varshney et al., 2017). Similarly, there is an expanded family of ABA transporters genes, which are involved in the transport of secondary metabolites associated with some cuticle components (Debieu et al., 2017). In addition, significant SNPs associated with aerial biomass was found to be near predicted gene Pgl_GLEAN_1003415; a putative 3-ketoacyl-CoA synthase (Debieu et al., 2018), that is known to be an essential condensing step in wax and suberin biosynthesis (Gable et al., 2004; Liu et al., 2014; Punnuri et al., 2017). While the effect of cuticle thickness and composition on the drought resistance in pearl millet has not been studied, it has been reported that pearl millet is less prone to nanoparticle toxicity at germination and rooting stage because of its thicker cuticle (Jain et al., 2017). In addition, the leaf surfaces of pearl millet accessions resistant to downy mildew were found to have significantly thicker cuticle and an increase in wax content (Chalam, 1996). Further work needs to be done to establish the role of cuticle thickness and composition on water loss, both before and after stomatal closure.

#### 2.2.3 Asynchronous tiller development

Plasticity in tiller development is a characteristic of both pearl millet and its wild progenitors (Vadez et al., 2012), with primary tillers developing in the axils of leaves of the main culm and secondary tillers from buds in the axils of leaves on the primary tillers. These tillers transition to flowering at different times, depending on their age. This is called asynchronous tillering and can allow plants to recover from drought by developing tillers that flower after the dry spell has ended (Craufurd and Bidinger, 1988). A mid-season drought that terminates at or prior to flowering causes a significant increase in tiller development, which later can fully compensate for grain number and yield losses in the main culm inflorescence (Mahalakshmi et al., 1987). However, drought loss caused during the grain filling period of pearl millet with terminal drought stress cannot be effectively compensated by this trait (Mahalakshmi et al., 1987), possibly because those tillers initiated and commencing growth under drought stress are unlikely to be productive (Mahalakshmi et al., 1988). The genetic control of tiller number in pearl millet has only been scantily studied, concentrating on the ortholog of the transcriptional regulator, Teosinte Branched 1 (TB1) and showing that it could have been involved in domestication (Remigereau et al., 2011). There are also QTL studies for variation in panicle number, which directly measures productive tillers that produce an inflorescence (**Table 1**) (Yadav et al., 2002). QTL analyses have been performed for stover and biomass yield which are related to both tiller number and growth vigor (Yadav et al., 2019). More research should be focused on the asynchronous tiller development of pearl millet as these tillers have the potential to influence yield and allow the plant to recover from the effects of drought. In addition, further work on the timing of initiation and fate of tillers in response to environmental stimuli is likely to present novel targets for selection, in addition to TB1.

#### 2.2.4 Flowering plasticity

Early flowering is an important drought escape mechanism in plants, and in wheat, early flowering after a relatively short vegetative period is a response for impending conditions of terminal stress (Shavrukov et al., 2017). Pearl millet is a short-day plant, but large genotypic differences exist in the photoperiodic requirement for flowering among pearl millet varieties depending upon the zone of latitude that they are adapted to. Almost 54.4% of total cultivated pearl millet germplasms have been found to flower irrespective of the day length, although most are facultatively photoperiod sensitive, showing a delay in flowering time with increasing daylength (Rai et al., 1999). Germplasms originating from equatorial Africa are usually strictly photoperiod sensitive, needing comparatively short days to flower. Landraces with early flowering (33–40 days) are predominantly found in Pakistan, Ghana, Togo, and India; with very late flowering (121–159 days) in Sierra Leone and the Central African Republic (Upadhyaya et al., 2007). Pearl millet grown under long day length together with midseason water stress was found to delay its flowering time, but did not cause a significant effect on grain yield (Mahalakshmi et al., 1987). They suggested that later floral initiation is a drought escape mechanism adapted by pearl millet in early mid-season droughts, just as early floral initiation is adaptive for late-season drought. Varieties are adapted to time flowering close to the end of the rainy season, ensuring completion of maturation with remaining soil moisture (Vadez et al., 2012). However, climate change involving changes in rainfall patterns may mean that formerly adapted landraces no longer flower in sync with local conditions. QTL associated with change in flowering time under drought stress have been found on LG2, colocalized with a major drought tolerant QTL (**Tables 1**, **S1**). Co-localized genes included transcription factors belonging to known flowering time gene families, including zinc finger CCCH type and MADS-box gene families (Sehgal et al., 2012).

## 3 Grain yield under drought stress

Many genetic analyses have targeted variation in grain yield in pearl millet, which is a combination of number of productive tillers, size of the inflorescence, fertilization success, and effectiveness of seed development and maturation. The effect of drought on yield can affect any or all of these traits, but most trials have focused on only the overall effect on grain yield variation of these traits under drought conditions. We have summarized the results of all the QTL studies conducted in pearl millet till date in **Tables 1**, **S1**. The first QTL associated with yield and drought tolerance traits in pearl millet under drought conditions were identified in a hybrid mapping population created from the parental lines H 77/833-2 and PRLT 2/89-33 that differed in response to drought (Yadav et al., 2002). A genetic map of 50 markers, comprising both restriction fragment length polymorphism (RFLP) and microsatellite (SSR) markers, was used in the analysis, and QTLs were identified for terminal drought tolerance imposed when 50% of the population had commenced flowering. Such a treatment targets the grain-filling stage of seed maturation. Drought tolerance index for superior grain yield was mapped to genomic regions on linkage 1 and 2, with changes seen in increased panicle number and harvest index (ratio of grain yield to vegetative biomass) (**Tables 1**, **S1**). In another study, hybrid genotypes were subjected to moderate to severe terminal drought stress environments, where there was a consistent grain yield advantage for hybrids carrying alleles from drought tolerant genotypes in both drought and well-watered environments (Serraj et al., 2005). Along with this, a fine mapping approach has been taken for identifying genes under validated drought-QTL on linkage group 2 where various expressed sequence tag based markers have been identified (Sehgal et al., 2012). Similarly, fine mapping of various traits associated with canopy development, water use, biomass production and crop production under drought stress have been done within these region (Tharanya et al., 2018). Yadav et al. (2004) using the same parents but a wider range of moisture environments than they had previously used (Yadav et al., 2002), found that linkage group 2 had a significantly higher grain yield effect in all moisture regimes, and related this to adaptation to varying moisture environments during the grain filling stage. In addition to finding QTL on linkage group 2, two additional QTLs (LG3 and LG4) were identified as the primary candidates for marker assisted selection to improve grain yield across different moisture conditions of drought stress (Bidinger et al., 2007).

## 4 Insight into pearl millet drought tolerant mechanisms using transcriptomics

The recent availability of the pearl millet draft genome has accelerated the transcriptomic study of drought stress tolerance (Varshney et al., 2017). Five papers have reported transcriptomics study on drought stress in pearl millet (Choudhary and Padaria, 2015; Dudhate et al., 2018; Jaiswal et al., 2018; Shivhare et al., 2020a; Shivhare et al., 2020b). The earliest transcriptomic studies exploring the genes responsible for drought tolerance were done using the suppressive subtraction hybridization technique on seedlings exposed to various stresses, including salt, drying, and cold (Mishra et al., 2007), and 30% polyethylene glycol 600 (Choudhary and Padaria, 2015). Many of the differentially expressed genes (DEGs) were shared between the two studies and includes genes that regulate the expression of genes in response to stresses, such as transcription factors, and those that protect cellular structures and prevent denaturation of proteins and enzymes, such as *late embryogenesis abundant (LEA)* genes. Similarly, genes like *heat shock protein (HSP)*, proteases, transporters, including aquaporins, detoxification genes to deal with the enhanced production of reactive oxygen species under stress conditions, and genes involved in enhanced proline production were also found in both studies.

Recent studies on gene expression dynamics under drought stress have primarily been accomplished by RNA-seq analyses on plants at both the vegetative and reproductive stages. At vegetative stages, roots showed more differentially expressed genes than leaves, and both tissues showed DEGs associated with abscisic acid and proline accumulation, as well as upregulation of HSP dehydrins, and *LEA* genes (Jaiswal et al., 2018). A similar study by Dudhate et al. (2018), compared drought susceptible and tolerant pearl millet genotypes and found that gene expression was much more affected in the drought tolerant line (~four times more upregulated DEGs and ~ten times more down-regulated DEGs) than in the sensitive line. Shivhare et al. (2020a) also examined response to terminal drought stress in a drought tolerant and a drought sensitive line at the reproductive stage, and most of the DEGs from tolerant genotypes were reported to be associated with secondary metabolite pathways such as mevalonate, shikimate, alkaloids, phenols, flavonoids, lignin, and wax biosynthesis, and stress associated phytohormones such as ABA, ethylene, gibberellic acid, jasmonic acid and salicylic acid. Multiple DEGs were associated with Photosystem I and II with upregulation in the drought tolerant genotype, suggesting that electron transport in PS I and PS II might be affected by drought stress in tolerant genotypes. Shivhare et al. (2020a) also examined drought stress in a drought tolerant line at vegetative (25 days after sowing) and reproductive stage (40 days after sowing) and found higher secondary metabolite production at reproductive versus vegetative stages, suggesting that pearl millet invests more resources into secondary metabolite production at flowering time to protect the most vulnerable part of the plant.

**Figure 2:**
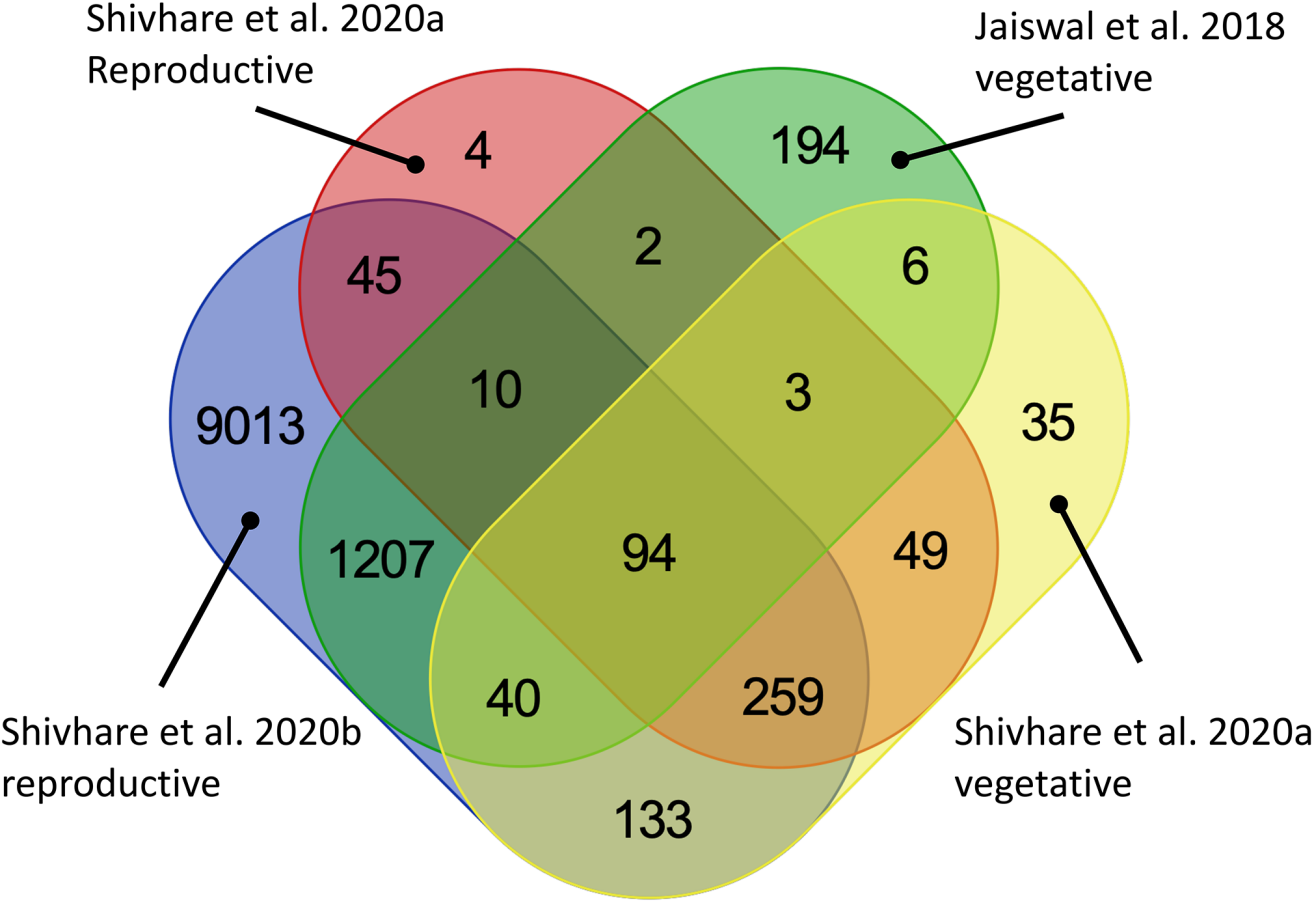
Venn diagram showing shared differentially expressed genes across four published transcriptomic datasets. The datasets differed in the conditions examined: Shivhare A at 40 DAS (Shivhare et al., 2020b) compares a drought tolerant genotype with a drought sensitive genotype under drought stress. The other three datasets [Jaiswal vegetative (Jaiswal et al., 2018) and Shivhare B vegetative and reproductive (Shivhare et al., 2020a)] compare drought tolerant genotypes under well-watered versus drought stressed conditions.

**Figure 3:**
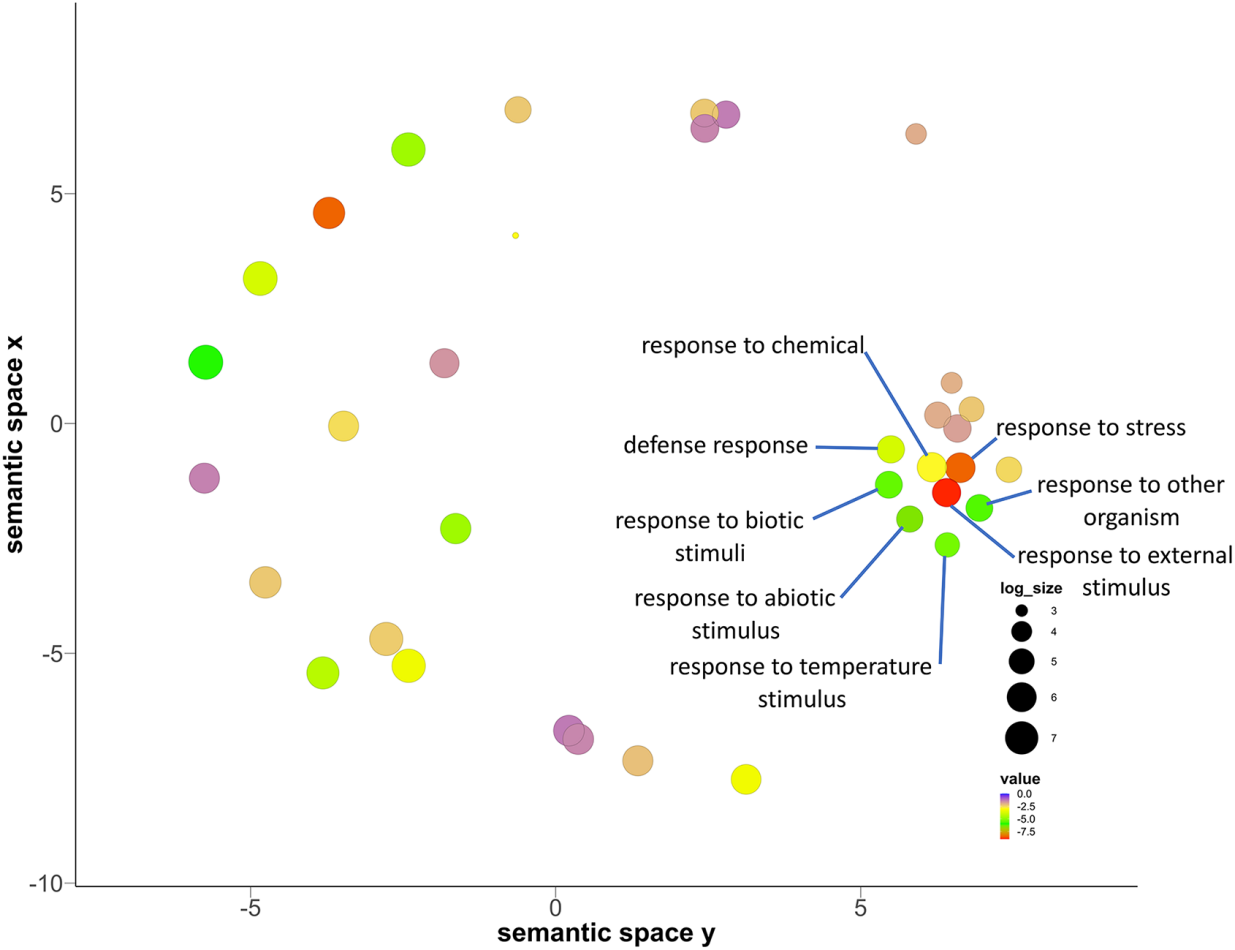
Biological process related gene ontology (GO) enrichment analysis of the 94 genes common to all studies that were compared, presented in a multidimensional scaling-based visualization from REVIGO. Out of 28 main GO terms, the GO terms with semantic similarities (genes having similar ontology are compared based on degree of relatedness by the similarity in meaning of their annotations) have clustered together. Clusters are represented by most significant GO term found by REVIGO.

These five studies provide extensive transcriptomic evidence to support the genetic and physiological basis of drought tolerance in pearl millets, yet the variety of accessions, methods, and aims make it difficult to draw comparisons between them. We therefore decided to conduct a combined analysis of three of these studies (Jaiswal et al., 2018; Shivhare et al., 2020a; Shivhare et al., 2020b), that had used comparable, next-generation sequencing-based techniques, and with their sampling strategies covering both vegetative and reproductive stages (**Table 2**). One of these studies compares a drought tolerant versus a drought sensitive genotype under drought stress (Shivhare et al., 2020a) while the other three compare a tolerant genotype with or without drought stress. We also realized that gene annotation in the current pearl millet genome assembly draft is still relatively limited and insufficient for our purpose, so, as a compromise, we converted pearl millet genes to their Arabidopsis homologs using blastp and took advantage of the extensive available Arabidopsis annotation resources. This strategy allowed us to interrogate gene expression differences across studies, even though the annotation may not be completely specific. Our methodology, in brief, was to extract differential expressed genes from the three pearl millet datasets and BLAST them against Arabidopsis protein sequences (TAIR10) using blastp (with e-value < 10^-5^). Common shared genes were then selected and visualized by Venn diagrams (https://bioinformatics.psb.ugent.be/webtools/Venn/) to ascertain the intersection of datasets, and a Gene Ontology (GO) enrichment analysis was conducted by agriGO (http://systemsbiology.cpolar.cn/agriGOv2/#:~:text=for%20Agricultural%20Community-,AgriGO%20v2.,the%20realm%20of%20ontology%20analyses, Du et al. 2010) and visualized by REVIGO http://revigo.irb.hr/, (Supek et al., 2011). The intersection of the DEG lists from the datasets comparing well-watered and drought stress treatment effects on a tolerant genotype identified 97 shared genes (**Figure 2A**) whereas the comparison of all datasets identified 94 shared genes between all stages (**Figure 2B**; **Table S2**). REVIGO-visualized GO enrichment analysis of the 94 shared genes displayed a discrete cluster associated with abiotic stress separated from the other genes (**Figure 3**; **Table S2**). Among these genes, 25 genes closely associated to drought were studied in detail for their role and functions (**Table 3**).

**Table 2:** Summary of published transcriptomics data used in our study with detailed information on methodology and data sets.

Plant material	Growth stage at which drought stress was applied and part of tissue collected	Transcriptomics analysis method	Threshold and level of fold change considered for DEG	Blast e-value	References
Drought tolerant pearl millet J-2454	29 Days after sowing (leaf tissues but non-specific part mentioned)	Trinity platform (*de novo* assembly)	FDR< 0.05, 2.0	10^-3^	Jaiswal et al., 2018
PLRT2/89-33 (tolerant) and H77/833-2 (sensitive)	40 Days after sowing (flag leaf)	Trinity platform (*de novo* assembly)	FDR< 0.05, 2.0	10^-5^	Shivhare et al., 2020b
PLRT2/89-33 (tolerant)	25 Days (upper most leaf) and 40 DAS after sowing (flag leaf)	Assembly using reference genome	FDR< 0.05, 2.0	10^-5^	Shivhare et al., 2020a

**Table 3:** Detailed functions of shared genes from transcriptomics data. The function of genes is from original papers not just from TAIR database.

Gene ID	Pearl millet ID and expression	TAIR Gene name	Functions based on reference reports	References
AT1G53540	Pgl_GLEAN_10029349/ upregulate	*Heat shock* *protein (HSP)*	It functions as molecular chaperones of newly synthesized proteins, preventing their accumulation over time during thermal stress to maintain them in a state competent for ATP dependent as well as independent refolding	Wu et al., 2019
AT5G25610	Pgl_GLEAN_10010984/ upregulate	*Responsive to* *dehydration 22 (RD22)*	*RD* genes are mediated by abscisic acid (ABA), is known to be induced under salt stress and drought stress in Arabidopsis.	Yamaguchi-Shinozaki and Shinozaki, 1993
AT1G32080	Pgl_GLEAN_10021397/ upregulate	*Plastidal glycolate* */glycerate translocator1 (PLGG1)*	*PLGG1* deficient plants were simultaneously affected ABA responses, which includes ABA-mediated stomatal movement and stress tolerance.	Dong et al., 2018
AT5G57560	Pgl_GLEAN_10038433/ upregulate	*Calmodulin and* *calmodulin related* *genes*	The regulation of this calmodulin-related gene family in Arabidopsis suggests that calcium ions and calmodulin are involved in transduction of signals from the environment, enabling plants to sense and respond to environmental changes.	Braam and Davis, 1990
AT3G04720	Pgl_GLEAN_10029185/ upregulate	*Pathogenesis* *related genes (PR-4)*	In Arabidopsis, *PR-4* gene which showed positive roles against necrotrophs and showed less expression following 10hr cold treatment.	Mishina and Zeier, 2007
AT2G07050	Pgl_GLEAN_10026807/ upregulate	*Cycloartenol* *synthase 1 (CAS1).*	The *CAS1* mutants Arabidopsis showed abnormal growth of leaves and arrest of shoot and root meristems which is probably the consequence of sterols depletion. Sterols play a prominent role in abiotic stress.	Babiychuk et al., 2008
AT3G12500	Pgl_GLEAN_10032140/ upregulate	*Pathogenesis* *related genes 3 (PR-3) Chitinase*	*PR-3* are basic endochitinase and are involved in jamsonic/ethylene signaling pathway. The PR-3 genes were slightly upregulated in Arabidopsis during wounding.	Chandrashekar et al., 2018
AT5G24530	Pgl_GLEAN_10000809/ upregulate	*Downy mildew resistant 6 (DMR6)*	*DMR6* encodes salicylic acid 5-hydroxylase that fine tunes salicylic acid homeostasis. SA plays essential roles in biotic and abiotic responses, plant development and leaf senescence.	Zhang et al., 2017
AT4G02380	Pgl_GLEAN_10028629/ upregulate	*Late Embryogenesis* *Abundant 5 (LEA5)*	Increases the tolerance to oxidative stress and showed increased root growth and shoot biomass in H2O2 stressed condition.	Karpinska et al., 2022
AT1G79460	Pgl_GLEAN_10012752/ upregulate	*Ent-kaurene* *synthase (KS1)*	*KS1* encodes enzymes of mevalonic acid biosysnthesis pathway. Many isoprenoids in the MVA pathway play important roles as mediators of interactions between plants and their environment, such as defense responses against biotic and abiotic stresses.	Caelles et al., 1989; Cho et al., 2022
AT3G46970	Pgl_GLEAN_10032453/upregulate	*Alpha-glucan* *phosphorylase 2 (PHS2)*	*PHS2* mutant with knock out of these gene develop leaf lesions when placed in very low light and leaves senescence occurs very rapidly and important for prolonged development when carbohydrate levels are unbalanced.	Schopper et al., 2015
AT2G03200	Pgl_GLEAN_10020803/upregulate	*Atypical aspartic* *Protease in roots 1* *(ASPR1)*	*ASPR1* overexpression mutants display shorter primary roots and a pronounced reduction in the number of lateral roots in Arabidopsis. *ASPR1* emerges as the first AP with an anticipated role in the regulation of root development in Arabidopsis.	Soares et al., 2019
AT1G15910	Pgl_GLEAN_10030568/upregulate	*Factor of DNA* *methylation 1 (FDM1)*	Belongs to a subgroup of *SGS3*-like proteins that act redundantly in RNA-directed DNA methylation. DNA methylation is associated with transcriptional silencing.	Xie et al., 2012
AT1G05805	Pgl_GLEAN_10008692/upregulate	*ABA-responsive* *kinase substrate 2* *(AKS2)*	*AKS*2 transcribed genes encoding potassium channels that promoted stomatal opening*. AKS*-mediated transcription stimulates stomatal opening, a process that is antagonized by ABA-induced phosphorylation of *AKS* transcription factors.	Takahashi et al., 2013
AT3G57520	Pgl_GLEAN_10008593/downregulate	*Seed imbibition* *2 (SIP2)*	*SIP2* encodes a raffinose-specific alpha-galactosidase that catalyzes the breakdown of raffinose into alpha-galatose and sucrose. This enzyme may function in unloading raffinose from the phloem as part of sink metabolism.	Peters et al., 2010
AT2G39770	Pgl_GLEAN_10005886/ downregulate	*Cytokinesis* *defective 1 (CYT1)*	Encodes a *GDP*-mannose pyrophosphorylase/mannose-1-pyrophosphatase. This enzyme provides *GDP*-mannose, which is used for cell wall carbohydrate biosynthesis and protein glycosylation as well as for ascorbate biosynthesis. Mutations in this gene confer hypersensitivity to NH4+.	Barth et al., 2010
AT4G11650	Pgl_GLEAN_10033680/ downregulate	*Osmotin* *34 (OSM34)*	Osmotin-like protein; functions as a positive regulator in the generation of ABA responses and is under post-translational control	Park and Kim, 2021
AT5G24090	Pgl_GLEAN_10022544/ downregulate	*Chitinase A (CHIA)*	Chitinase A expressed exclusively under environmental stress conditions. Shown be a plant lysozyme involved in plant immunity.	Żur et al., 2013
AT5G20410	Pgl_GLEAN_10031058/ downregulate	*Monogalactosyldiacyl* *Glycerol synthase 2* *(MGD2)*	In Arabidopsis, *MGD2* mutants under aluminum stress showed severe root inhibition, plasma membrane integrity damage and lipid peroxidation.	Liu et al., 2020
AT3G45140	Pgl_GLEAN_10020461/ downregulate	*Lipoxygenase 2 (LOX2)*	In Arabidopsis, *LOX2* is involved in the generation of oxylipins during senescence induced by both endogenous as well as exogeneous factors such as pathogen attack, drought, salt stress etc. It also impacts the accumulation of JA.	Seltmann et al., 2010
AT5G45820	Pgl_GLEAN_10036265/ downregulate	*CBL-interacting* *protein kinase 20 (CIPK20)*	*CBL* mutant become hypersensitive to ABA treatment in Arabidopsis. The *CBL9* mutant plants showed enhanced expression of genes involved in ABA signaling, such as ABA-INSENSITIVE 4 and 5. *CBL-CIPK* module modulates the ABA repressor in Arabidopsis during seed development.	Sanyal et al., 2017
AT2G39050	Pgl_GLEAN_10007409/ downregulate	*ArathEULS3 (EULS3)*	Transcript levels for *ArathEULS3* increased after exposure to ABA and osmotic treatments. *ArathEULS3* in Arabidopsis conferred ABA hypersensitivity and enhanced drought tolerance	Li et al., 2014
AT5G10230	Pgl_GLEAN_10012413/ downregulate	*Annexin 7 (ANN7)*	It was found to response to cold, heat, salt stress and water deprivation, played important role in secretive pathways which involves ATPase and peroxidase activity	Clark et al., 2001
AT4G21510	Pgl_GLEAN_10015794/ downregulate	*F-box stress* *Induced 2*	F-box overexpression induced cell division in stomata formation in Arabidopsis producing large clusters in mutants which had defects in stomata. Moreover, it causes the increase in stomatal density.	Li et al., 2021
AT1G65680	Pgl_GLEAN_10009578/ downregulate	*Expansin B2 (EXPB2)*	Arabidopsis *EXPB2* mutant showed reduced cell wall-bound peroxidase activity and decreased oxidative stress tolerance. *EXPB2* are cell wall proteins that induces cell wall loosening.	Han et al., 2015

The cluster associated with temperature stimuli included GO terms such as defense response, response to chemical, response to abiotic stimulus, response to biotic stimulus, response to organic substance and cellular response to stimulus (**Figure 3**; **Table S2**). We found several categories of encoding genes were assigned to these GO terms. *HSP17.6C*, a member of the *heat shock protein (HSPs)* family was found to be upregulated in the tolerant genotype under drought stress. In Arabidopsis, *HSP17.6* expression was found to be proportional to the severity of heat stress, leading to six-fold increase during abrupt heat stress (Wu et al., 2019). It functions as a molecular chaperone for newly synthesized proteins, preventing their accumulation over time during thermal stress, so as to maintain them in a state competent for ATP dependent as well as independent refolding (Queitsch et al., 2000; Al-Whaibi, 2011; Yamaguchi et al., 2021). Heat shock proteins are also known to be expressed under a wide range of stresses such as osmotic, salinity, oxidative, desiccation, and heavy metal stresses (Ghatak et al., 2016). Another shared gene, *Responsive to dehydration 22 (RD22)* was found to be upregulated in the tolerant genotype under drought stress. *RD* genes are mediated by abscisic acid (ABA), and are known to be induced under salt stress and drought stress in Arabidopsis (Yamaguchi-Shinozaki and Shinozaki, 1993), especially early in the stress response, as the *RD22* mRNA begins to appear within 2hr of the start of dehydration and is strongly expressed by 5hr in *Arabidopsis* seeds (Yamaguchi-Shinozaki and Shinozaki, 1993).

The important stress gene, *LEA*, is hydrophilic and improves the plant’s capacity to retain water during drought (Banerjee and Roychoudhury, 2016). This gene was found to be upregulated in the tolerant pearl millet genotype under drought stress. Arabidopsis plants that overexpressed *AtLEA5* showed tolerance to oxidative stress and overexpression of *AtLEA5* in barley lead to increased seed yield (Karpinska et al., 2022). *LEA5* regulates organellar translation, to enhance respiration relative to photosynthesis in response to stress (Karpinska et al., 2022). Introduction of *LEA* protein genes into spring wheat also significantly increased water use efficiency compared to non-transgenic lines under water deficit conditions (Sivamani et al., 2000). Another shared gene is the calcium binding protein, annexin, which in Arabidopsis was found to respond to cold, heat, salt stress and water deprivation, and played an important role in secretive pathways that involved ATPase and peroxidase activity (Clark et al., 2001). In *Arabidopsis*, loss of function mutants of *Annexin* showed greater sensitivity to drought stress while gain of function mutants caused an increase in resistance (Konopka-Postupolska et al., 2009). The annexin family has been found to decrease ROS production, hence reducing oxidative stress and preventing cell death (Clark et al., 2001). In rice, the annexin gene family has been shown to be involved in various abiotic stresses including heat, cold, drought and salinity (Jami et al., 2012).

The other GO annotation groups include genes related to carbohydrate metabolism, terpenoid biosynthesis, secondary metabolic process, immune system process and response to stimulus (**Figure 3**; **Table 3**). The carbohydrate metabolism includes genes related to GO terms such as proteolysis, lipid metabolism, primary metabolism, and carboxylic metabolism. Carbohydrate related biosynthesis genes like *monogalactosyldiacylglycerol synthase 2* (*MGD2*) were downregulated in the tolerant pearl millet. In Arabidopsis, *MGD2* mutants under aluminum stress showed severe root inhibition, plasma membrane integrity damage and lipid peroxidation (Liu et al., 2020). The genes relating to the terpenoid synthesis family, *cycloartenol synthase 1 (CAS1)* were upregulated in the tolerant pearl millet genotypes during drought stress. *CAS1* initiates sterol biosynthesis by converting 1,3-oxidosquaelene to cycloartenol (Babiychuk et al., 2008). The *cas1* mutants of Arabidopsis showed abnormal growth of leaves and arrest of shoot and root meristems, which is probably the consequence of sterol depletion (Babiychuk et al., 2008). Sterols are integral components of the membrane lipid bilayer in plants where they regulate membrane fluidity, which influence its structure, properties and functions. Plant membranes are affected by various environmental conditions and sterols play a prominent role in plant response to abiotic stress (Rogowska and Szakiel, 2020).

**Figure 4:**
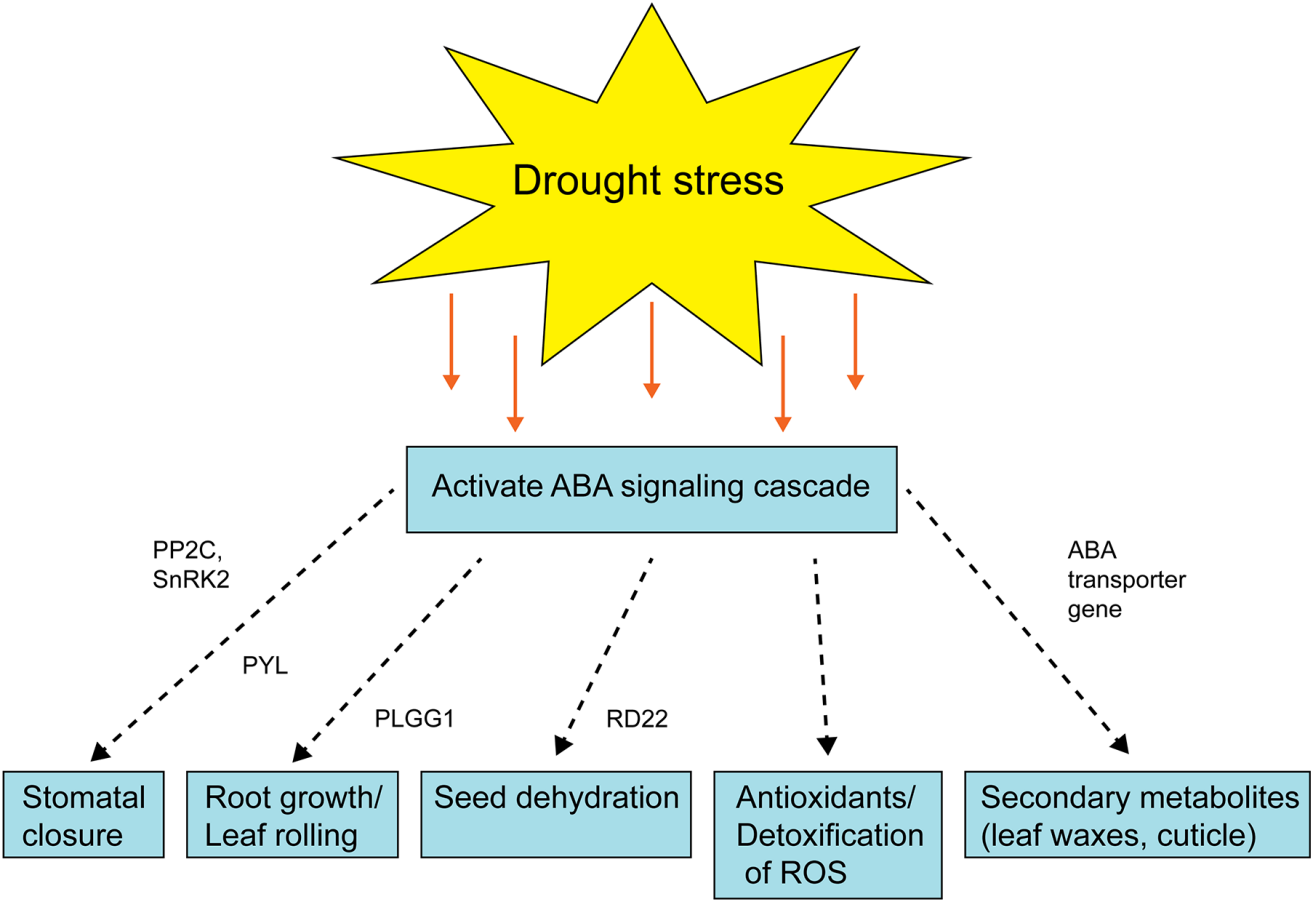
Schematic diagram showing the role of ABA signaling after drought stress activation. The dashed arrow represents the multiple steps are involved and green box represents the enzyme/gene for that process. PYL, pyrabactin resistance (PYR)-like; PP2C, *protein phosphatases*; SnRK2s, *SNF1-related* protein kinase; PLGG1, *plastidal glycolate/glycerate translocator 1*; RD22, *Responsive to dehydration 22*.

There were several clusters enriched in GO terms related to secondary metabolites and phytohormone pathways, including *plastidal glycolate/glycerate translocator 1 (PLGG1)* whose response to abscisic acid is upregulated in the tolerant pearl millet genotype under drought stress. In *Arabidopsis*, a mutant with a knockout of *PLGG1* showed growth defects and reduction in photosynthetic rates (South et al., 2017). Similarly, *PLGG1* deficient Arabidopsis showed enhanced sensitivity to ABA-mediated stomatal movement and drought tolerance (Dong et al., 2018). In addition, *PLGG1* regulates lateral root growth under abscisic acid treatment (Dong et al., 2018) Abscisic acid is a mainly produced in plant roots under drought stress as it progressively increases hydraulic connectivity and stimulates root elongation during water-limiting conditions (Daszkowska-Golec, 2016). The plants with elongated roots will regulate water use efficiency to delay or avoid osmotic stress (Dong et al., 2018). Another phytohormone gene, *CBL interacting protein kinase (CIPK20)*, is downregulated in tolerant pearl millet genotypes under drought stress. In Arabidopsis, *CBL* mutants become hypersensitive to ABA treatments, with *CBL9* mutant plants showing enhanced expression of genes involved in ABA signaling such as ABA-insensitive 4 and 5 (Pandey et al., 2004). ABA is a key player under drought stress and its broad role is summarized in **Figure 4**. In addition to ABA-related genes, genes related to biotic stress, *pathogenesis-related 3* (*PR-3*) and *PR-4*, were upregulated in tolerant pearl millet genotypes during drought. In Arabidopsis, the *PR-4* gene has a role in protection from necrotrophs and cold treatments (Wu et al., 2019). Similarly, *PR-3* which is involved in the jasmonic/ethylene signaling pathway, is upregulated during wounding (Chandrashekar et al., 2018). *Pathogenesis-related* genes are regulated by SA production, which will ultimately induce systemic acquired resistance in plants (Kesarwani et al., 2007). However, the specific links between these defense genes and abiotic stress still needs further study.

The important genes for recurring drought known as memory genes are important for drought as they are repetitive, and memory genes enables plants to respond to the subsequent drought by altering the expression patterns of the responsive genes. These memory genes respond more promptly and strongly to the repeated drought stress (Ding et al., 2012; Kim et al., 2020). The research on Arabidopsis plants showed that plants trained with subsequent dehydration events wilted much slower than the non-trained plants (Ding et al., 2012). Similarly, priming (pre-exposure to mild drought) in wheat before anthesis improved tolerance to drought stress after anthesis and increased the grain yield in comparison to non-primed plants under drought stress (Wang et al., 2014). These primed or trained plants have memory marks like histone modifications through methylation, chromatin remodeling, microRNAs, accumulations of proteins, phytohormones, and growth regulators in inactive form to efficiently modulate gene expression in subsequent stress (Jacques et al., 2021). As all the transcriptomics studies used in our combined analysis were devoid of recurring drought experiments so far or were destructive studies even if the studies were done at two different time points, it was not feasible to pinpoint the memory genes among the common gene. However, some overlapped genes were associated with memory genes like functions. A gene named *Factor of DNA methylation 1* (*FDM1*) belonging to SG33-like protein family which act redundantly in RNA directed DNA methylation was found to be upregulated in tolerant pearl millet genotype in drought stress (Xie et al., 2012). Mainly it is known that DNA methylation plays key roles in maintaining genome stability, modulation of gene expression and transcriptional silencing (Moazed, 2009) but recent findings suggest that DNA methylation might contribute drought memory formation in rice (Kou et al., 2022) as most of the memory transcripts expressed during stress were significantly associated with DNA methylation changes (Li et al., 2019). However, the drought memory genes seemed to be species specific and there has not been studies done in pearl millet to identify drought memory transcripts. Hence, future experiments on identifying the memory genes in pearl millet will be of great interest to breeders as it will help improve the plants to adapt to fluctuating environments for longer term.

## 5 Conclusions and perspectives

Understanding the morphological, physiological, and genetic basis of the drought resistance mechanisms in pearl millet is crucial for helping farmers in marginalized areas, as they rely heavily on pearl millet for subsistence farming. These insights into the drought resistance mechanisms of pearl millet may well be useful for improving other cereal crops that also face increased drought stress caused by shifting climates. In this review we have partitioned drought resistance strategies in pearl millet into shorter- and longer-term responses. Short term responses such as stomatal conductance and osmotic adjustment are major strategies used by pearl millet to tolerate drought. The long-term drought response of deep rooting and asynchronous tiller production are traits of pearl millet that have created a crop that has enhanced developmental plasticity to adapt to changing environmental conditions. However, the underground parts have received less attention in comparison to aboveground parts due to difficulty in phenotyping. It is crucial to understand the genetics behind the root hydraulic traits and architecture of pearl millet, which can be used as a model for drought root response. Asynchronous tiller development, a trait of pearl millet which is used as both drought escape and as a recovery trait during vegetative intermittent drought stress, allows plants to develop tillers and flower at different times and help to fully compensate mid-season droughts. Tillering and flowering plasticity are important traits that can be exploited for changing climates, however more integrative studies that link genetics, genomics, phenomics and physiology should be performed in these two traits.

The transcriptomics results support the drought resistance strategies of tolerant genotypes of pearl millet in both seedling and post vegetative stages. The 94 common genes which were shared between three datasets at vegetative and reproductive stage show a distinct cluster for stress related genes which need to be further studied to understand drought resistance. Further, to support these several DEGs of drought resistance, proteomics and metabolomics study should be performed in pearl millet. The important challenge here will be to efficiently explore these -omics data sets and find the key functional genes and pathways related to drought resistance. In addition, designing experiment to identify the memory genes will be rewarding to all the plant breeders as it will help to improve the long-term cereal crop adaptation to this fluctuating environment.

In summary, pearl millet combines multiple strategies that makes it major drought tolerant cereal crop. This crop provides many avenues that can be explored to understand and be used to improve drought resistance in other cereal crops. Pearl millet has huge diversity which are adapted to diverse latitude and rainfall pattern, that provides an opportunity to explore these drought traits further. However, there are key questions that researchers and breeders need to consider, especially the targets of selection. A major desirable characteristic of pearl millet for farmers in the poorest conditions is the ability for tillers to take over some of the yield burden from the main culm, should intermittent water stress affect the main culm. Reducing tillering is a main objective for most breeding programs as it allows for larger yield from just a few stems, but this may not be desirable for a crop that promises flexibility in the face of climatic uncertainty. Understanding both near-term genetic consequences of water stress as well as the pathways that allow recovery should both be aims pursued by breeders. We suggest that future transcriptomic studies target tiller buds, inflorescences, and rooting tips, so that researchers can better understand the trade-offs at play in the response of this crop to drought. In addition, drought resistance in pearl millet should be evaluated with recent tools and in association with other major abiotic stresses, such as high temperature and salinity because drought stress occurs along with these stresses and crosstalk occurs between stresses at various levels.

## Data availability statement

Publicly available datasets were analyzed in this study. This data can be found here: PRJNA385901 for Jaiswal et al., 2018, and list of DEGs in **Supplementary Materials** PRJNA574746 for Shivhare et al., 2020a, and list of DEGs in **Supplementary Materials** PRJNA607164 for Shivhare et al., 2020a, and list of DEGs in **Supplementary Materials**.

## Author contributions

NS and AD conceived the idea of this review, NS and HH performed the transcriptomics analysis, KS, NS, and HH performed the GO analysis and researched the functions of individual genes, and all contributed to the writing and editing of the manuscript. All authors contributed to the article and approved the submitted version.
